# “Resuscitation on the go”- Musculoskeletal discomfort, CPR quality and provider experiences in a transit ambulance setting: A simulated, mixed-methods study

**DOI:** 10.1186/s12245-026-01365-0

**Published:** 2026-09-10

**Authors:** Caroline Ashritha Maben, Prithvishree Ravindra, Abraham Samuel Babu, Mohammad Khalid, B. N. Lavanya, Ankit Kumar Sahu, Vimal Krishnan S

**Affiliations:** 1 Present Address: Department of Trauma Care Management, Yenepoya School of Allied Health Sciences, Yenepoya (Deemed to be University), Mangalore, Karnataka India; 2 Department of Emergency Medical Technology, Manipal College of Health Professions, Manipal Academy of Higher Education, Manipal, India; 3 Department of Emergency Medicine, Kasturba Medical College, Manipal Academy of Higher Education, Manipal, India; 4 Department of Physiotherapy, Manipal College of Health Professions, Manipal Academy of Higher Education, Manipal, India; 5 Higher Colleges of Technology, Abu Dhabi, UAE; 6 Department of Emergency Medicine, All India Institute of Medical Sciences, New Delhi, India

**Keywords:** Cardiopulmonary resuscitation, Ambulances, Emergency medical services, Musculoskeletal pain, Postural balance, Ergonomics

## Abstract

**Background:**

Performing high-quality cardiopulmonary resuscitation (CPR) in a moving ambulance poses biomechanical and ergonomic challenges. This pilot study aimed to evaluate musculoskeletal discomfort, CPR quality, and provider posture, while exploring the experiences of healthcare providers delivering CPR in a moving ambulance.

**Methods:**

A mixed-methods study involving 30 Emergency Medical Service (EMS) providers and students aged 18–40 years trained in Basic Life Support was conducted. Participants performed CPR simulation in an in-hospital stationary setting and a moving ambulance using a Little Anne^®^ QCPR mannequin (Laerdal Medical, Stavanger, Norway). Musculoskeletal discomfort was assessed before and after CPR in the moving ambulance using the Numerical Pain Rating Scale. CPR quality parameters, including compression score, depth, rate, recoil, and chest compression fraction, were recorded using the QCPR^®^ app. Body posture was assessed using mean trunk and elbow angles during compression and recoil. Semi-structured interviews explored participants’ experiences.

**Results:**

Median pain scores increased significantly after CPR in the moving ambulance [0 (0, 2.75) before vs. 4 (0, 6) after; *p* < 0.001]. The most common pain sites were the lumbar region and wrists. Chest compression score and good rate percentage were lower in the moving setting. Trunk and elbow angles showed greater flexion in the moving ambulance, with changes observed across consecutive CPR cycles. Qualitative analysis revealed themes of physical fatigue, balance issues, safety concerns, and ergonomic limitations. Participants recommended simulation-based training, improved ambulance design, and mechanical CPR devices.

**Conclusion:**

CPR in a moving ambulance was associated with increased musculoskeletal discomfort and lower CPR quality metrics. Ergonomic modifications, provider training, and mechanical aids may help address these challenges. Further studies with larger and more diverse populations and real-world transport conditions are warranted.

**Trial registration:**

Prospectively registered in Clinical Trial Registry India (CTRI/2024/09/073482) on September 5th, 2024.

**Supplementary Information:**

The online version contains supplementary material available at https://doi.org/10.1186/s12245-026-01365-0.

## Background

Despite significant advances in resuscitation science, survival to hospital discharge following out-of-hospital cardiac arrest (OHCA) remains low [1]. Emergency Medical Services (EMS) play a critical role in the chain of survival through early recognition of cardiac arrest, delivery of high-quality cardiopulmonary resuscitation (CPR), early defibrillation, and timely transport to definitive care. The annual incidence of EMS-treated OHCA ranges from 30.0 to 97.1 per 100,000 population [2]. Although rapid transport is an integral component of prehospital care, performing CPR during ambulance transport presents unique operational and clinical challenges for EMS providers.

High-quality CPR is essential for improving survival and favourable neurological outcomes following cardiac arrest. Although ambulances facilitate the continuation of prehospital resuscitation during transport, maintaining high-quality CPR while ensuring provider safety presents considerable operational and ergonomic challenges [3, 4]. Prehospital CPR may be interrupted during patient loading, rhythm analysis, and defibrillation, potentially compromising CPR quality [5]. Furthermore, the no-flow fraction, which is the proportion of time during CPR when chest compressions are interrupted, is reported to be higher during ambulance transport than under stationary conditions [6]. EMS providers often perform chest compressions from unsecured standing or seated positions within the confined ambulance workspace, where stretcher height and limited working space may influence compression quality [7]. These challenges are further compounded by the operational conditions of ambulance transport, including vehicle movement and environmental factors such as road conditions, which may affect both CPR performance and provider safety [8]. 

In addition to these challenges, rescuer fatigue may be intensified by the physical demands of maintaining stability while performing chest compressions. The confined space within an ambulance may require EMS providers to adopt constrained working postures, potentially increasing musculoskeletal strain and discomfort [6, 9]. Performing chest compressions while standing without adequate support may also increase the risk of loss of balance and provider injury.

Although previous studies have examined CPR performance during ambulance transport, limited evidence is available on the combined assessment of provider musculoskeletal discomfort, CPR quality, body posture, and provider experiences during transport CPR. The effects of the confined ambulance workspace and operational conditions of transport on provider comfort and performance have also not been adequately characterized. Objective assessment of provider posture using body-angle analysis, together with qualitative exploration of providers’ immediate experiences following transport CPR, may provide additional insight into the ergonomic and operational challenges encountered during resuscitation in transit.

Therefore, this pilot mixed-methods simulation study aimed to evaluate musculoskeletal discomfort, CPR quality, body posture, and provider experiences during CPR performed under simulated transit ambulance conditions. Specifically, the study assessed musculoskeletal discomfort experienced by EMS providers during CPR in a moving ambulance, compared CPR quality and body posture between stationary in-hospital and moving ambulance settings, and explored provider experiences related to comfort, safety, and challenges encountered during transport CPR through qualitative interviews.

## Materials and methods

The study was reported in accordance with the Strengthening the Reporting of Observational Studies in Epidemiology (STROBE) guidelines for the quantitative component (Supplemental Table 1).

### Study setting and participants

The study was conducted in accordance with the principles of the Declaration of Helsinki at a tertiary care university teaching hospital in South India, following approval from the Institutional Ethics Committee (IEC2 454/2024). This mixed-methods study was conducted using a simulated CPR protocol under two conditions: a stationary in-hospital setting within the Department of Emergency Medicine and a moving ambulance setting. Participants included EMS students and EMS providers aged 18–40 years who had received Basic Life Support training. The majority of participants were female (90%), reflecting the available study population during the recruitment period from August 2024 to December 2024 (Supplemental Table 2). Participants were excluded if they reported a diagnosed herniated intervertebral disc, recent musculoskeletal injury, or previous musculoskeletal surgery. Written informed consent was obtained from all participants. The study was prospectively registered with the Clinical Trials Registry–India (CTRI/2024/09/073482).

The sample size was estimated based on the primary outcome of musculoskeletal discomfort. Assuming a prevalence of 50%, an absolute precision of 18%, and a 95% confidence interval, the minimum required sample size was calculated as 30 participants. The study was not specifically powered to detect differences in CPR quality metrics.

### Study procedure

#### CPR simulation in an in-hospital setting

**Fig. 1:**
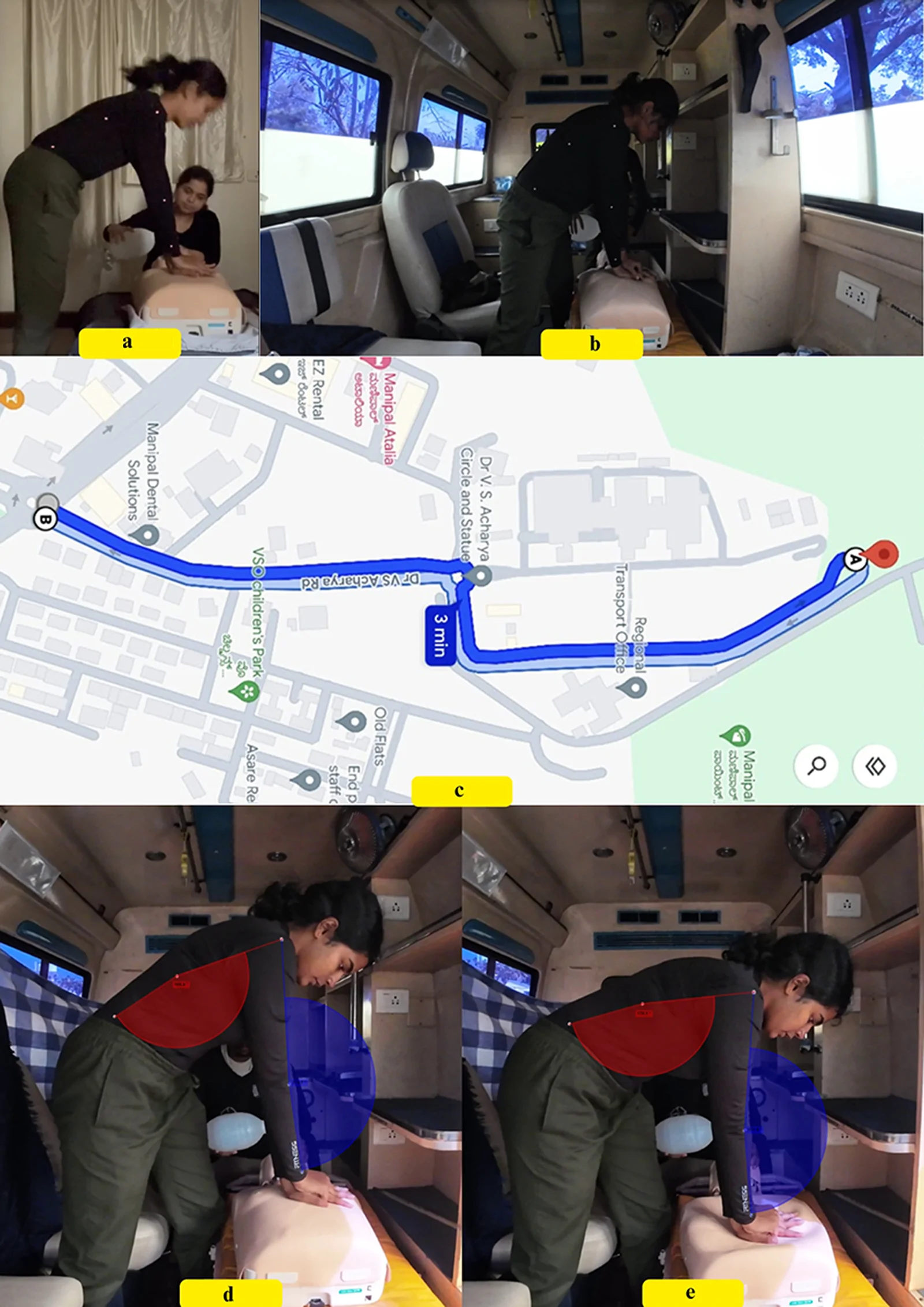
Details of study methodology. **a** Reflective marker position during CPR simulation in in-hospital setting; **b** Reflective marker position during CPR simulation in a moving ambulance setting; **c** Route map utilized for CPR performed in a moving ambulance (Image source – Google maps); **d** Kinovea software (version 2023.1.2; Kinovea Open Source Project, Bordeaux, France) interface showing body angle analysis during chest compression (Trunk and elbow angle); **e** Kinovea software (version 2023.1.2; Kinovea Open Source Project, Bordeaux, France) interface showing body angle analysis during chest recoil (Trunk and elbow angle)

The CPR simulation in the stationary condition was conducted within the Department of Emergency Medicine. The bed height was adjusted to approximately 43 cm to match the stretcher height in the Advanced Life Support ambulance, allowing comparison of provider posture between the two conditions. To facilitate video-based posture assessment, participants wore black, full-sleeve shirts, and 6-mm reflective markers were placed at five anatomical landmarks: the lateral border of the acromion, greater trochanter, lateral condyle of the humerus, ulnar styloid process, and posterior midaxillary line at the T12 level, as shown in Fig. 1a. Participants worked in pairs. One participant performed chest compressions on a Little Anne^®^ QCPR manikin (Laerdal Medical, Stavanger, Norway) for 2 min while the other provided bag-mask ventilation, after which they switched roles and continued CPR for a further 2 min. CPR was recorded using a GoPro^®^ HD action camera (GoPro Inc., San Mateo, CA, USA) positioned approximately 380 cm from the participants for subsequent posture analysis.

#### CPR simulation in a moving ambulance setting

Before the moving ambulance simulation, participants were assessed for the presence and location of musculoskeletal pain using a body chart and, when pain was reported, its intensity was recorded using the Numerical Pain Rating Scale (NPRS). Participants worked in the same pairs in both the stationary and moving conditions. The ambulance stretcher was fixed at a height of 43 cm from the ambulance floor. CPR was recorded using a GoPro^®^ HD action camera (GoPro Inc., San Mateo, CA, USA) positioned approximately 380 cm from the participants. The camera was held by an investigator seated at the rear of the ambulance, with the CPR provider maintained at the centre of the recorded frame. Participants wore black, full-sleeve shirts, and reflective markers were placed at the same anatomical landmarks used in the stationary condition (Fig. 1b).

A predefined route was used for all moving ambulance simulations (Fig. 1c), incorporating turns, speed bumps, rough road segments, and smooth road segments to represent varied road conditions. The ambulance was driven at a maintained speed of 30–40 km/h throughout the simulations. Sudden acceleration and braking were avoided, and sharp turns were minimized to reduce the risk of participant injury. The same route and driving conditions were maintained across simulations. Each pair performed 12 min of CPR, alternating between chest compressions and bag-mask ventilation every 2 min.

#### Qualitative interview

Following the moving ambulance simulation, semi-structured interviews were conducted using a validated interview guide (Supplementary Material) to explore participants’ experiences. Interviews were audio-recorded and subsequently transcribed for analysis. The interview domains included overall experience, perceived CPR quality during ambulance transport, provider comfort and safety, and recommendations for improving transport CPR.

### Outcome measures

Musculoskeletal discomfort was assessed using a body diagram and the Numerical Pain Rating Scale (NPRS). Provider experiences were explored using the qualitative interview guide. CPR quality was assessed using the QCPR^®^ app (Laerdal Medical, Stavanger, Norway), which provided chest compression score, mean compression depth, percentage of compressions achieving adequate depth, mean compression rate, percentage achieving the target rate, percentage with adequate release, and chest compression fraction. The compression score is a composite metric developed by Laerdal Medical that incorporates chest compression depth, rate, and recoil [10]. 

Video recordings were analysed using Kinovea software (version 2023.1.2; Kinovea Open Source Project, Bordeaux, France) to assess provider posture. For each participant, trunk and elbow angles were measured at two points during each chest compression: maximum chest compression and maximum chest recoil, as illustrated in Fig. 1d and e. Within each 2-minute CPR cycle, 15 compressions were selected for analysis: five from the beginning of the cycle, five at approximately 1 min, and five toward the end of the cycle. These time points were selected to characterize changes in posture across the CPR cycle while maintaining the feasibility of detailed video analysis. Mean trunk and elbow angles were calculated from the 15 selected compressions for each cycle. For comparison between conditions, the overall mean of the three cycles in the moving ambulance was compared with the mean angle measured in the stationary setting.

### Statistical analysis

**Fig. 2:**
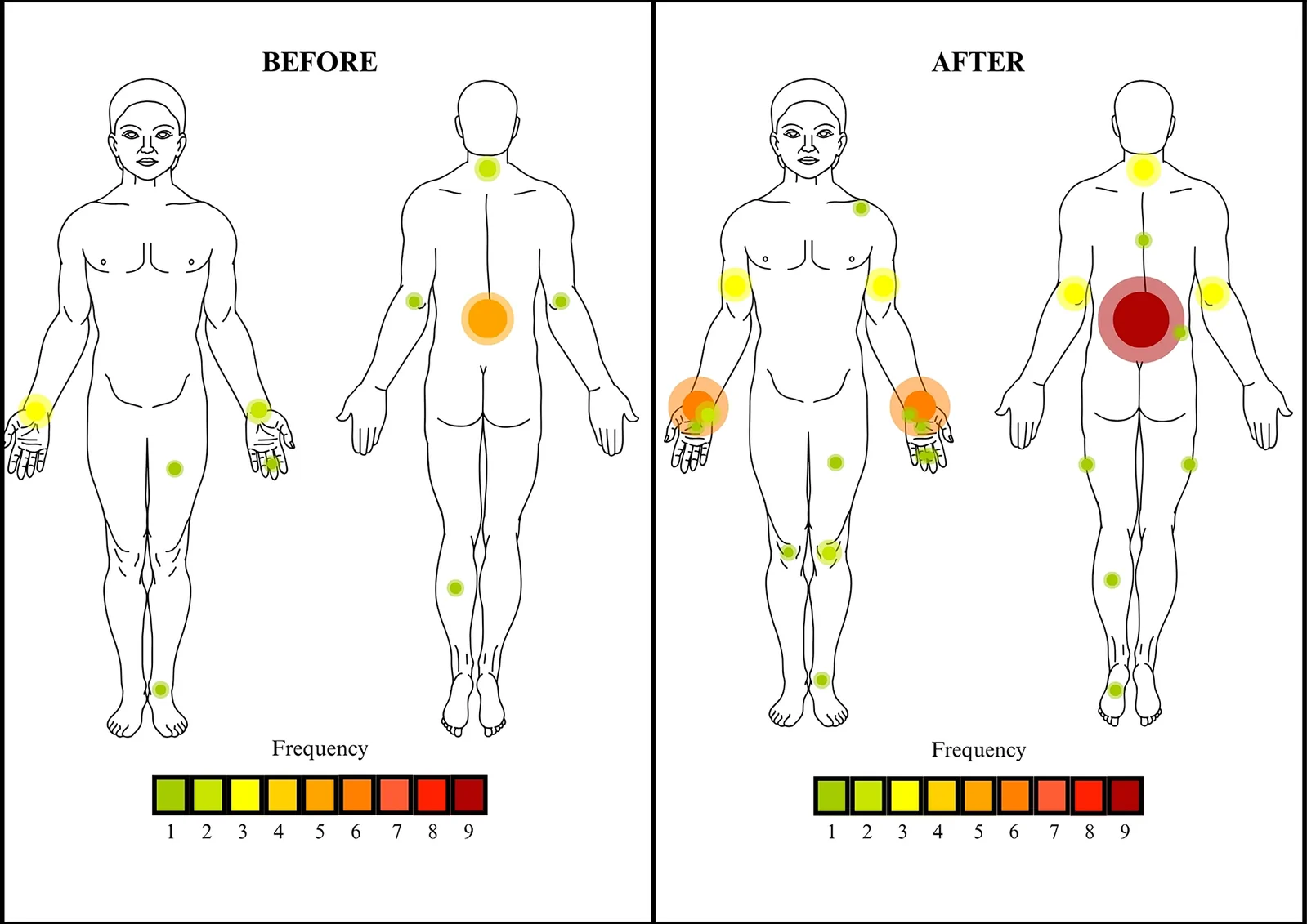
Body map showing common body regions with pain reported before and after CPR simulation in a moving ambulance. The size and colour are indicative of frequency (number of participants reporting pain in the body region) alone, not the intensity of pain in the affected areas

Statistical analysis was performed using Jamovi software (version 2.4.11; The Jamovi Project, Sydney, Australia). Descriptive statistics were used to summarize participant characteristics. A change in pain score was defined as an increase of ≥ 2 points. The Wilcoxon signed-rank test was used to compare NPRS scores before and after CPR in the moving ambulance. The frequency and distribution of pain across body regions before and after CPR were visualized using a heat map generated in Canva (version 1.73.0.0; Canva Pty Ltd., Sydney, Australia) (Fig. 2). CPR quality metrics were assessed for distribution and summarized using median and interquartile range. Trunk and elbow angles were summarized as mean and standard deviation.

Audio-recorded interviews were transcribed verbatim and analysed using an iterative thematic analysis approach. The research team independently reviewed transcripts, generated initial codes, grouped related codes into categories, and developed overarching themes through discussion and consensus. Dedoose software (version 10.0.23; SocioCultural Research Consultants, LLC, Los Angeles, CA, USA) was used to facilitate code organization and retrieval.

## Results

A total of 30 participants were included in the study, with a mean age of 21.77 ± 1.3 years; 27 (90%) were women. All participants completed the CPR simulation in both the stationary in-hospital and moving ambulance settings.

### Musculoskeletal discomfort

The median (25th–75th percentile) maximum NPRS score increased from 0 (0, 2.75) before CPR to 4 (0, 6) after CPR in the moving ambulance setting. This increase was statistically significant on the Wilcoxon signed-rank test (*p* < 0.001). The most frequently reported site of pain was the lumbar region (*n* = 9), followed by the wrists (*n* = 6), and the cervical region, elbow joints, and arms (*n* = 3 each) (Fig. 2).

### Quality of CPR

CPR was performed by all participants in both the stationary in-hospital and moving ambulance settings. As shown in Table 1, the median (25th–75th percentile) chest compression score was higher in the stationary setting than in the moving ambulance setting: 90 (87.5, 97) versus 71 (68, 81.5), respectively. The percentage of compressions delivered at the recommended rate was also lower in the moving ambulance setting than in the stationary setting (65% vs. 89%).

**Table 1:** Comparing CPR Metrics between in-hospital setting and moving ambulance setting (*N* = 15 pairs)

	Stationary (In-hospital setting)		Moving (Ambulance setting)	
	Median	Q1-Q3	Median	Q1-Q3
Compression Score	90	87.5–97	71	68–81.5
Good Release in %	99	97–100	97	94.5–97.5
Adequate Depth in %	99	98–99	97	97–98
Mean Depth (cm)	7.1	6.35–7.25	7.3	7.05–7.3
Average Rate per min	109	107–112	116	113–120.5
Good Rate %	89	50.5–98	65	38–81
Chest Compression Fraction	82	80.5–83	83	80–84.5

### Body posture

**Fig. 3:**
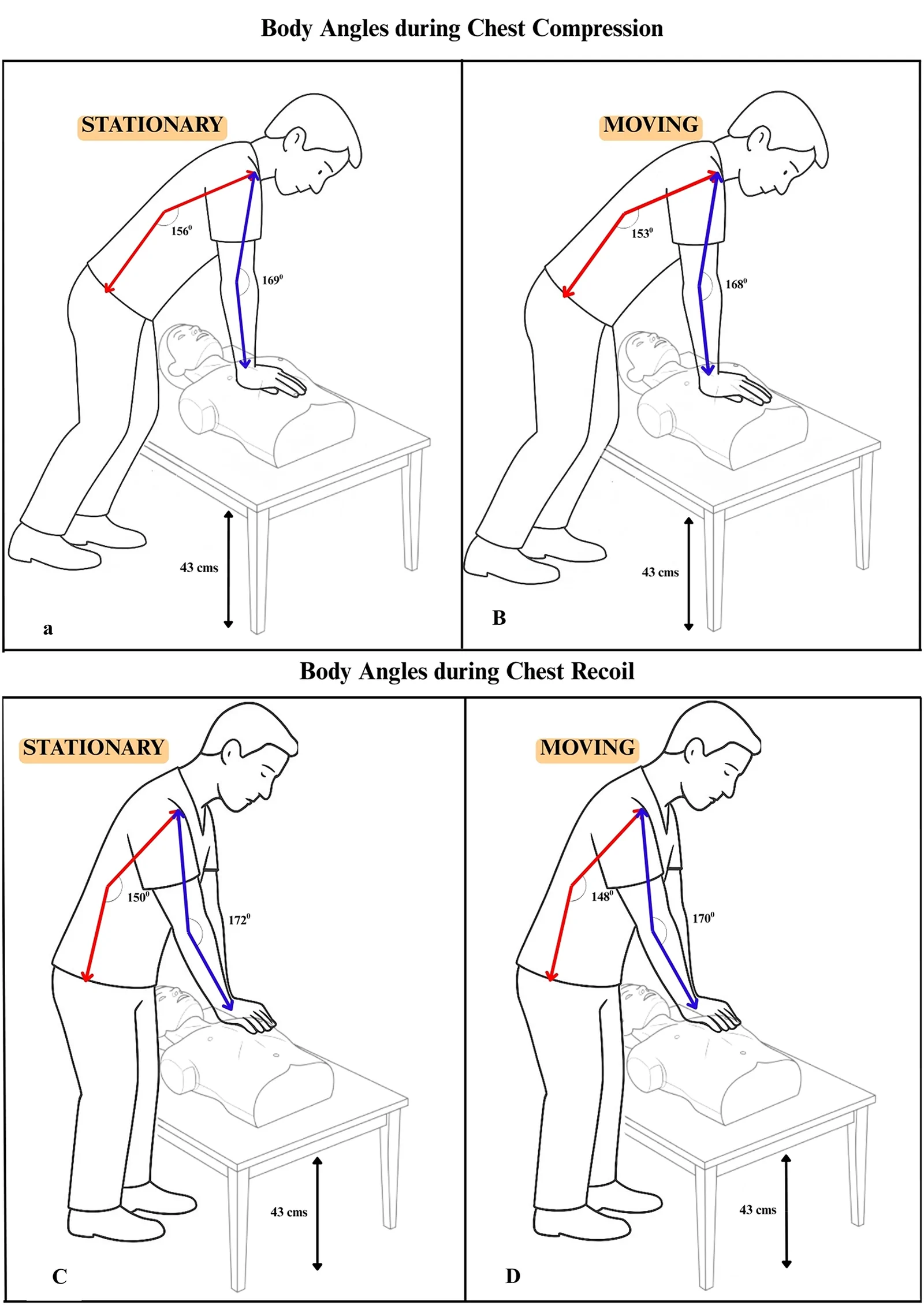
Mean trunk and elbow angles during chest compression and chest recoil in a simulated CPR setting. **a** Chest compression in an in-hospital setting; **b** Chest compression in a moving ambulance setting; **c** Chest recoil in an in-hospital setting; **d** Chest recoil in a moving ambulance setting

The mean trunk and elbow angles during chest compression and recoil showed greater flexion in the moving ambulance condition than in the stationary condition, with mean differences of approximately 2–3° for trunk angle and 1–2° for elbow angle (Fig. 3). Across the three CPR cycles performed in the moving ambulance, the measured angles approached those observed in the stationary condition by the third cycle (Table 2).

**Table 2:** Trend in mean trunk and elbow angles from stationary CPR to 3 cycles of moving CPR

	Condition	Mean ± SD	95% CI
Trunk Angle during Chest Compression	Stationary	156.08 ± 11.65	151.73–160.43
	Moving − 1st cycle	151.79 ± 12.28	147.20–156.37
	Moving − 2nd cycle	153.59 ± 13.47	148.56–158.62
	Moving − 3rd cycle	154.24 ± 13.70	149.13–159.35
Trunk Angle during Chest Recoil	Stationary	150.47 ± 12.85	145.67–155.27
	Moving − 1st cycle	147.05 ± 11.63	142.71–151.40
	Moving − 2nd cycle	148.06 ± 12.11	143.54–152.58
	Moving − 3rd cycle	148.50 ± 12.79	143.72–153.27
Elbow Angle during Chest Compression	Stationary	169.01 ± 7.80	166.09–171.92
	Moving − 1st cycle	167.59 ± 10.24	163.77–171.41
	Moving − 2nd cycle	167.66 ± 10.26	163.83–171.50
	Moving − 3rd cycle	169.17 ± 11.44	164.90–173.44
Elbow Angle during Chest Recoil	Stationary	171.94 ± 7.11	169.29–174.60
	Moving − 1st cycle	170.02 ± 8.40	166.89–173.16
	Moving − 2nd cycle	170.45 ± 8.03	167.45–173.45
	Moving − 3rd cycle	171.17 ± 10.01	167.43–174.91

### Provider experiences

**Fig. 4:**
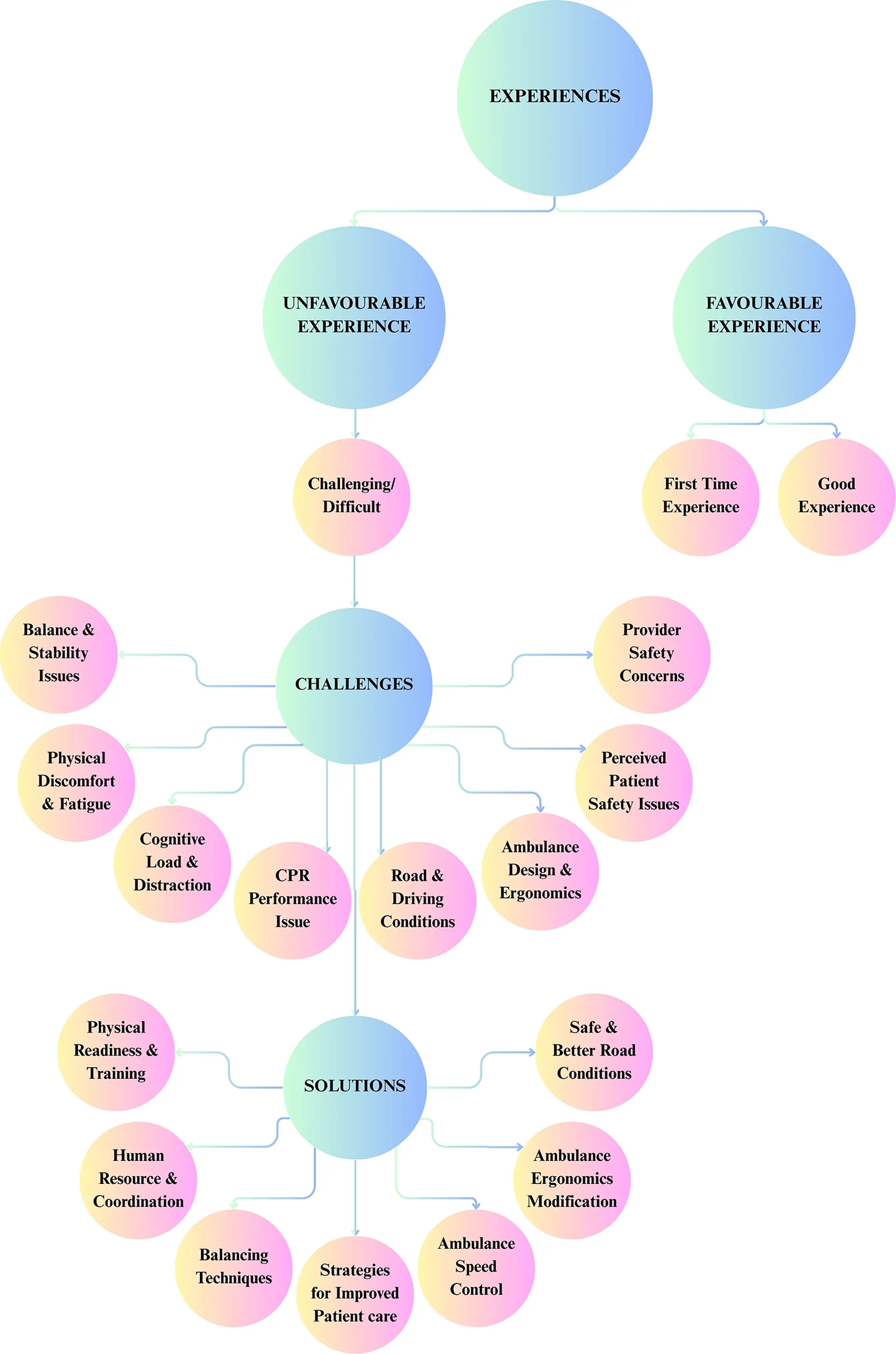
Visual representation of the thematic analysis of qualitative interview regarding provider experiences on performing CPR simulation in a moving ambulance

All participants were interviewed following completion of CPR in the moving ambulance to explore their experiences during transport CPR. Their experiences are summarized in Fig. 4 and detailed in Supplemental Table 3. The major themes that emerged included overall experience (“It was too difficult as it was a moving ambulance”; P03), physical discomfort and fatigue (“Continuous strain with concentration makes me feel dizzy. It gets me exhausted easily”; P13), balance and stability (“It is kind of really difficult to balance yourself in a moving ambulance when you give CPR”; P01), cognitive load and distractions (“In the ambulance, in front of us, there is a rack to keep the cardiac monitor and everything else. We have a fear that we might hit our head against that”; P04), perceived challenges in delivering high-quality CPR (“In the moving ambulance, we can’t achieve all the five components of high-quality CPR. There will be interruptions and no adequate chest recoil”; P18), road and driving conditions (“Because of the speed, we were falling forward on the mannequin, and it was very difficult for us to hold the position”; P13), ergonomics (“When we switch between ventilation and giving CPR, to move was a challenge”; P05), provider safety (“While giving CPR I hit my head twice”; P02), and perceived patient safety (“In moving ambulance, patient also will be moving”; P16). Further details are provided in Supplemental Table 3.

When participants were asked about strategies to improve the delivery of high-quality CPR during ambulance transport, themes emerged in two broad areas: (a) improving CPR quality (Supplemental Table 4) and (b) improving provider safety and comfort (Supplemental Table 5). Suggestions for improving CPR quality included physical readiness and simulation-based training (“More simulations in the moving ambulance, so that we get an idea of how to do it”; P30), human resource and team coordination (“At least 2 paramedics are required to coordinate and perform CPR”; P02), strategies to improve balance (“We can use some techniques such as widening our legs to keep balance”; P19), use of mechanical CPR devices (“Well, one thing is CPR device, which can be used to have consistent CPR performance”; P09), improved road conditions and ambulance stability (“Other way we can improve the quality is if the roads are good”; P15), and improved ambulance design (“We have less space inside. So, more space is needed for us to stand comfortably”; P10). For provider safety and comfort, participants suggested strategies to stabilize the provider (“If we have some belt or something to buckle, we will not lose balance and move around”; P12) and modifications to ambulance interiors to promote safety, such as padding or smoothing potentially hazardous edges (“The compartments and the edges could be padded or have a smooth edge”; P01).

## Discussion

Performing CPR in a moving ambulance presents biomechanical and operational challenges for EMS providers. In this study, participants reported a significant increase in musculoskeletal pain following CPR in the moving ambulance, with the lumbar region and wrists being the most frequently reported sites of discomfort. These findings are consistent with previous research reporting musculoskeletal discomfort among ambulance personnel following CPR, with 60% (191/318) reporting back discomfort after every episode of CPR and 45% (143/318) reporting discomfort involving the wrists, shoulders, and knees [11]. The present findings add to this evidence by describing musculoskeletal discomfort in the context of a simulated moving ambulance environment and by combining pain assessment with CPR quality, posture analysis, and qualitative exploration of provider experiences.

CPR may contribute to musculoskeletal discomfort through repetitive and forceful movements during chest compressions, including sustained forward flexion required to achieve adequate compression depth [12]. In the ambulance environment, these demands may be compounded by the confined working space and the need to maintain stability during vehicle movement. Previous studies have reported increased low-back movement and compression forces when CPR is performed on a low surface, which may contribute to back discomfort [9]. Rescuer position has also been associated with musculoskeletal discomfort; Foo et al. reported lower back pain when CPR was performed in a kneeling position compared with a standing position [13]. However, adopting a kneeling position may not always be feasible within the restricted space of an ambulance.

The current study identified greater mean trunk and elbow flexion during chest compressions and recoil in the moving ambulance compared with the stationary in-hospital setting. These differences may reflect changes in body positioning associated with the confined working environment and the need to maintain stability during vehicle movement. The angles approached those observed in the stationary condition by the third cycle of CPR. Although this pattern may indicate a change in posture over time, the study design does not allow us to determine whether this represented postural adaptation, fatigue-related postural change, adjustment to the CPR technique, or random variation. During CPR, the upper body functions as a closed kinematic chain, with force transmitted through the shoulders and upper limbs to the hands positioned over the sternum [14, 15]. Increased flexion of the elbows or trunk may increase reliance on upper-limb musculature and potentially contribute to fatigue and discomfort [16]. This may be one possible explanation for the upper-limb discomfort observed among participants in the present study.

The overall chest compression score and percentage of compressions delivered at the recommended rate were lower in the moving ambulance than in the stationary in-hospital setting. These findings are consistent with previous studies reporting that CPR quality may be compromised during ambulance transport [17–19]. Paramedics are often faced with a dilemma between focusing on high-quality CPR on the scene (‘stay and play’) and the need to rapidly transport for definitive care (‘scoop and run’), where advanced interventions such as extracorporeal CPR and primary coronary intervention are possible [20]. Several factors that may contribute to CPR quality during transport include off-balancing forces [21, 22], speed and driving pattern of the ambulance [8, 23], rescuer position [3], rescuer fatigue, and ambulance ergonomics including stretcher bed height [7]. These factors were also reflected in the qualitative interviews, where participants described challenges related to balance, movement, fatigue, and the confined ambulance environment.

The qualitative interviews provided several participant-generated suggestions for improving CPR delivery and provider safety during ambulance transport. Simulation-based training within EMS curricula, adequate staffing and team coordination, techniques to improve provider stability, greater working space around the patient, and optimization of stretcher and ambulance ergonomics were among the suggested strategies. Participants also identified mechanical CPR devices as a potential approach to maintaining more consistent chest compressions during transport. Previous studies have reported that mechanical CPR devices can provide consistent compression delivery and may reduce the need for rescuers to maintain a physically demanding position during transport, although evidence regarding their effect on survival and cost-effectiveness remains uncertain [24–27]. In resource-limited EMS systems, implementation may also be constrained by equipment costs, maintenance requirements, and the need for staff training. Therefore, mechanical CPR should be considered as a potential strategy rather than a definitive solution, and its role should be evaluated in the context of local resources and transport practices.

Participants described the simulation as both challenging and a valuable learning experience. The qualitative findings highlighted loss of balance during vehicle turns and on uneven road surfaces, as well as concerns regarding contact with overhead or surrounding ambulance structures. These findings support consideration of ergonomic modifications to the ambulance environment, including protective padding or smooth finishing of potentially hazardous edges and structures. Participants also suggested stabilization aids that could reduce provider movement while allowing an effective CPR position. Although seat-belt restraint systems may improve ambulance crew safety, previous research has reported lower CPR quality when CPR is performed from a secured seated position compared with an unsecured standing position [3]. Therefore, future ergonomic solutions should aim to balance provider stabilization and safety with the ability to maintain effective chest compressions [6]. 

This study highlights the challenges associated with providing CPR during ambulance transport, including musculoskeletal discomfort, reduced CPR performance measures, altered provider posture, and concerns regarding balance and safety. The combination of quantitative, biomechanical, and qualitative findings provides a multidimensional description of the provider experience during transport CPR. These findings may inform future research evaluating ambulance ergonomics, provider stabilization strategies, simulation-based training, and approaches to maintaining CPR quality during transport.

## Conclusion

This pilot study demonstrates that simulated CPR during ambulance transport was associated with increased musculoskeletal discomfort, changes in provider posture, and lower CPR quality measures compared with the stationary in-hospital setting. Participants also reported challenges related to balance, fatigue, confined working space, and provider safety. These findings highlight the need for further evaluation of ergonomic modifications, provider stabilization strategies, simulation-based training, and mechanical CPR as potential approaches to support safer and more effective CPR during ambulance transport. Further studies with larger and more diverse EMS populations, individual-level CPR quality data, and real-world driving conditions are needed to determine the independent effects of vehicle movement and ambulance ergonomics on CPR performance and provider safety.

### Limitations

This study has several limitations. First, musculoskeletal discomfort was not assessed immediately after CPR in the stationary in-hospital setting; instead, the pre-ambulance CPR NPRS assessment was considered the baseline for comparison with the post-ambulance CPR assessment. Therefore, the observed increase in pain cannot be attributed specifically to vehicle movement, as the potential contribution of CPR-related exertion and the confined ambulance environment cannot be separated. The immediate post-CPR assessment was performed to capture participants’ symptoms promptly and minimize recall bias. Second, individual-level CPR quality metrics could not be retrieved from the QCPR application. The analysis therefore assessed overall CPR performance rather than within-participant changes in CPR quality. CPR was performed as a team, with participants alternating between chest compressions and bag-mask ventilation; however, the inability to obtain individual-level data limits the statistical interpretation of the CPR quality findings. Third, the participant cohort was predominantly female (90%) and included both EMS students and EMS providers, which may limit generalisability to other EMS populations. The findings should therefore be interpreted cautiously and considered primarily representative of the study population. Fourth, this was a simulated study, and a predefined route with relatively limited traffic and a maintained speed of 30–40 km/h was used to ensure comparable conditions across participants. Although the route included different road surfaces, turns, and speed bumps, the controlled driving conditions may not fully represent real-world ambulance transport, where traffic, acceleration, deceleration, sudden braking, and unpredictable road conditions may vary. Finally, the study compared a stationary in-hospital setting with a moving ambulance setting and did not include a stationary ambulance condition. Consequently, the independent effects of vehicle movement and the confined ambulance environment or ergonomic constraints cannot be separated. The study was also not specifically powered to detect differences in CPR quality metrics, and the findings should therefore be considered exploratory.

## Supplementary Information

Below is the link to the electronic supplementary material.

Supplementary Material 1

## Data Availability

All data supporting the findings of this study are available within the paper and its Supplementary Information.

## Abbreviations

CPR: Cardiopulmonary Resuscitation
EMS: Emergency Medical Services
OHCA: Out-of-Hospital Cardiac Arrest
NPRS: Numerical Pain Rating Scale
QCPR: Quality CPR (Laerdal QCPR)
