# Supplementary material for: “Resuscitation on the go”- Musculoskeletal discomfort, CPR quality and provider experiences in a transit ambulance setting: A simulated, mixed-methods study
Source: Int J Emerg Med. 2026 Sep 10;19:228. doi: 10.1186/s12245-026-01365-0 (PMC13563694; doi:10.1186/s12245-026-01365-0)
Supplement: Supplementary file 1 [file 12245_2026_1365_MOESM1_ESM.docx]

**Supplemental Table 1: Strengthening the reporting of observational studies in epidemiology (strobe) guidelines**

|  | Item No. | Recommendation | Page No. |
| --- | --- | --- | --- |
| **Title and abstract** | 1 | (*a*) Indicate the study’s design with a commonly used term in the title or the abstract | 1, 2 |
|  |  | (*b*) Provide in the abstract an informative and balanced summary of what was done and what was found | 2-3 |
| Introduction | | | |
| Background/rationale | 2 | Explain the scientific background and rationale for the investigation being reported | 3-4 |
| Objectives | 3 | State specific objectives, including any prespecified hypotheses | 4 |
| Methods | | | |
| Study design | 4 | Present key elements of study design early in the paper | 4-5 |
| Setting | 5 | Describe the setting, locations, and relevant dates, including periods of recruitment, exposure, follow-up, and data collection | 4 |
| Participants | 6 | (*a*) *Cohort study*—Give the eligibility criteria, and the sources and methods of selection of participants. Describe methods of follow-up  *Case-control study*—Give the eligibility criteria, and the sources and methods of case ascertainment and control selection. Give the rationale for the choice of cases and controls  *Cross-sectional study*—Give the eligibility criteria, and the sources and methods of selection of participants | 5 |
|  |  | (*b*) *Cohort study*—For matched studies, give matching criteria and number of exposed and unexposed  *Case-control study*—For matched studies, give matching criteria and the number of controls per case |  |
| Variables | 7 | Clearly define all outcomes, exposures, predictors, potential confounders, and effect modifiers. Give diagnostic criteria, if applicable | 6-7 |
| Data sources/ measurement | 8* | For each variable of interest, give sources of data and details of methods of assessment (measurement). Describe comparability of assessment methods if there is more than one group | 6-7 |
| Bias | 9 | Describe any efforts to address potential sources of bias | 5 |
| Study size | 10 | Explain how the study size was arrived at | 5 |

| Quantitative variables | 11 | Explain how quantitative variables were handled in the analyses. If applicable, describe which groupings were chosen and why |  | 7-8 |
| --- | --- | --- | --- | --- |
| Statistical methods | 12 | (*a*) Describe all statistical methods, including those used to control for confounding |  | 7-8 |
|  |  | (*b*) Describe any methods used to examine subgroups and interactions |  |  |
|  |  | (*c*) Explain how missing data were addressed |  |  |
|  |  | (*d*) *Cohort study*—If applicable, explain how loss to follow-up was addressed  *Case-control study*—If applicable, explain how matching of cases and controls was addressed  *Cross-sectional study*—If applicable, describe analytical methods taking account of sampling strategy |  |  |
|  |  | (*e*) Describe any sensitivity analyses |  |  |
| Results | | | | |
| Participants | 13* | (a) Report numbers of individuals at each stage of study—eg numbers potentially eligible, examined for eligibility, confirmed eligible, included in the study, completing follow-up, and analysed |  | 5 |
|  |  | (b) Give reasons for non-participation at each stage |  |  |
|  |  | (c) Consider use of a flow diagram |  |  |
| Descriptive data | 14* | (a) Give characteristics of study participants (eg demographic, clinical, social) and information on exposures and potential confounders |  | 5 |
|  |  | (b) Indicate number of participants with missing data for each variable of interest |  |  |
|  |  | (c) *Cohort study*—Summarise follow-up time (eg, average and total amount) |  |  |
| Outcome data | 15* | *Cohort study*—Report numbers of outcome events or summary measures over time |  |  |
|  |  | *Case-control study—*Report numbers in each exposure category, or summary measures of exposure |  |  |
|  |  | *Cross-sectional study—*Report numbers of outcome events or summary measures |  | 8 |
| Main results | 16 | (*a*) Give unadjusted estimates and, if applicable, confounder-adjusted estimates and their precision (eg, 95% confidence interval). Make clear which confounders were adjusted for and why they were included |  | 8 |
|  |  | (*b*) Report category boundaries when continuous variables were categorized |  |  |
|  |  | (*c*) If relevant, consider translating estimates of relative risk into absolute risk for a meaningful time period |  |  |

| Other analyses | 17 | Report other analyses done—eg analyses of subgroups and interactions, and sensitivity analyses |  | 8-10 | |
| --- | --- | --- | --- | --- | --- |
| Discussion | | | | |  |
| Key results | 18 | Summarise key results with reference to study objectives |  | 10-13 | |
| Limitations | 19 | Discuss limitations of the study, taking into account sources of potential bias or imprecision. Discuss both direction and magnitude of any potential bias |  | 13 | |
| Interpretation | 20 | Give a cautious overall interpretation of results considering objectives, limitations, multiplicity of analyses, results from similar studies, and other relevant evidence |  | 13 | |
| Generalisability | 21 | Discuss the generalisability (external validity) of the study results |  | 10-13 | |
| Other information | |  | | |  |
| Funding | 22 | Give the source of funding and the role of the funders for the present study and, if applicable, for the original study on which the present article is based |  | 14 | |

**PROVIDER EXPERIENCES AFTER CPR SIMULATION IN A MOVING AMBULANCE**

**Qualitative interview guide**

1. How did you find the experience of delivering CPR in a moving ambulance?

a) Can you elaborate?

b) What factors do you think contributed to that experience?

2. How do you think we can improve quality of CPR during moving

ambulance?

3. How do you think we can enhance safety and comfort of the health care

providers during CPR in a moving ambulance?

**Supplemental Table 2: Characteristics of Interview Participants**

| **Sl No** | **ID** | **Current designation** | **Gender** | **Age** | **Experience (years)** |
| --- | --- | --- | --- | --- | --- |
| 1 | P01 | MSc. EMT | Female | 23 | 5.5 |
| 2 | P02 | MSc. EMT | Female | 23 | 5.5 |
| 3 | P03 | EMT Faculty | Female | 24 | 5.5 |
| 4 | P04 | EMT Faculty | Female | 22 | 5.5 |
| 5 | P05 | EMT Intern | Female | 22 | 3.5 |
| 6 | P06 | EMT Intern | Female | 22 | 3.5 |
| 7 | P07 | EMT Intern | Male | 21 | 3.5 |
| 8 | P08 | EMT Intern | Female | 22 | 3.5 |
| 9 | P09 | EMT Intern | Male | 21 | 3.5 |
| 10 | P10 | MSc. EMT | Female | 23 | 5.5 |
| 11 | P11 | EMT Intern | Female | 21 | 3.5 |
| 12 | P12 | MSc. EMT | Female | 23 | 5.5 |
| 13 | P13 | MSc. EMT | Female | 22 | 4.5 |
| 14 | P14 | MSc. EMT | Female | 22 | 4.5 |
| 15 | P15 | BSc. EMT | Female | 19 | 2.5 |
| 16 | P16 | BSc. EMT | Female | 20 | 2.5 |
| 17 | P17 | BSc. EMT | Female | 20 | 2.5 |
| 18 | P18 | BSc. EMT | Female | 21 | 2.5 |
| 19 | P19 | BSc. EMT | Female | 23 | 2.5 |
| 20 | P20 | BSc. EMT | Female | 20 | 2.5 |
| 21 | P21 | BSc. EMT | Female | 20 | 2.5 |
| 22 | P22 | BSc. EMT | Female | 22 | 3.5 |
| 23 | P23 | EMT Intern | Female | 21 | 3.5 |
| 24 | P24 | EMT Intern | Female | 21 | 3.5 |
| 25 | P25 | MSc. EMT | Female | 24 | 5.5 |
| 26 | P26 | EMT Intern | Female | 21 | 3.5 |
| 27 | P27 | MSc. EMT | Female | 23 | 5.5 |
| 28 | P28 | MSc. EMT | Female | 23 | 4.5 |
| 29 | P29 | EMT Staff | Male | 23 | 5.5 |
| 30 | P30 | BSc. EMT | Female | 21 | 2.5 |

**Supplemental Table 3: Participant Feedback on the Experience of Delivering CPR in a Moving Ambulance and Contributing Factors**

| **Sl No** | **Theme** | **Subtheme** | **Frequency (number of participants)** | **Quote** |
| --- | --- | --- | --- | --- |
| 1 | Overall experience | Challenging/ difficult | 22 | *“It was too difficult as it was a moving ambulance” –* P03 |
|  |  |  |  | *“It was a bit challenging" –* P10 |
|  |  | First time | 15 | *“This is my first-time giving CPR in the ambulance” –* P11 |
|  |  |  |  | *“This was my first-time giving CPR in the moving ambulance” –* P18 |
|  |  | Good learning experience | 8 | *“Actually, it was a very good experience” –* P07 |
|  |  |  |  | *“It was a great experience because it was my first-time giving CPR in ambulance” –* P02 |
| 2 | Physical discomfort and fatigue | Tiring | 7 | *“We got tired, initially we did not, but after 2- 4 minutes, we got tired”* – P02 |
|  |  |  |  | *“It was tiring”* – P17 |
|  |  | Dizziness | 4 | *“Continuous strain with concentration makes me feel dizzy. It gets me exhausted easily”* – P13 |
|  |  |  |  | *“After 5 minutes, I was feeling a little tired and dizzy”* – P25 |
|  |  | Suffocation | 3 | *“We faced a lot of Suffocation inside”* – P03 |
|  |  |  |  | *“It was very clustered, and we were not comfortable”* – P10 |
|  |  | Back pain | 2 | *“After continuous CPR, in the lower back I could feel little pain”* – P01 |
|  |  |  |  | *“After giving CPR continuously, I got little back pain”* – P02 |
| 3 | Balance and stability issues | Loss of balance, specifically during turns | 24 | *“It is kind of really difficult to balance yourself in a moving ambulance when you give CPR”* – P01 |
|  |  |  |  | *“During turns we could not get the balance to stand”* – P22 |
|  |  | Balanced due to low speed | 1 | *“I was able to balance and give CPR, there was no issue with that because of the speed which was not so high”* – P09 |
| 4 | Cognitive load and distractions | Fear of potential injuries from sharp edges/objects | 3 | *“I felt my head was going to hit the cabinet”* – P08 |
|  |  |  |  | *“In the ambulance, in front of us, there is a rack to keep the cardiac monitor and everything else. We have a fear that we might hit our head against that”* – P04 |
|  |  | Fear of potential fall | 3 | *“When the turn comes or during sudden break or jump, we were scared we will fall back”* – P21 |
|  |  |  |  | *“Constantly we were thinking that we might get hurt, or hit our head, or cut our hands, or fall down”* - P15 |
| 5 | High Quality CPR challenges | Compression rate & depth changes during turns and humps | 5 | *“During the bumps on road, we could not give enough depth of compression”* – P20 |
|  |  |  |  | *“The quality of CPR, compression rate, etc would differ in a moving ambulance from stationary*” – P24 |
|  |  | Increased switching time | 4 | *“The switching time will be more”* – P11 |
|  |  |  |  | *“The person giving breaths had to jump through the chair, to switch and become compressor. So, we wasted about 15 to 20 seconds just moving around. So that creates a lot of gaps to the CPR”* – P13 |
|  |  | Not able to attain enough depth | 3 | *“I was not able to follow proper compression depth of five to six centimetres”* – P03 |
|  |  |  |  | *“Quality wise comparatively couldn’t give the same depth as stationary”* – P27 |
|  |  | Difficulty in keeping track of compression and ventilation | 2 | *“Keeping a track of compression count is very difficult”* – P03 |
|  |  |  |  | *“It is very difficult to follow 30:2 ratio as well”* – P04 |
|  |  | Hand positioning displacement | 2 | *“It was a little bit hot, humidity inside was more and my hands started to sweat, so I could not get the proper grip”* – P26 |
|  |  |  |  | *“The hand was getting misplaced during the turn or sudden break or bump”* – P21 |
|  |  | Increased interruptions in moving ambulance | 3 | *“If we are falling, we will catch something because of fear of falling. During that time, interruptions will be there. I feel in moving ambulance, there will be more interruptions”* – P21 |
|  |  |  |  | *“When there were U-turns, we had to remove our hands. Continuous CPR was not being performed”* – P06 |
|  |  | Difficult in initial 2 mins cycle | 1 | *“In first cycle I was not stable as it was a moving ambulance, and I was also moving. After that I was comfortable in giving CPR”* – P21 |
|  |  | Difficult to give compression while balancing | 1 | *“Because of the moving ambulance, we were not able to maintain both the hands together”* – P29 |
|  |  | Not able to attain adequate chest recoil | 1 | *“In the moving ambulance, we can't achieve all the five components of high-quality CPR. There will be interruptions and no adequate chest recoil”* – P18 |
| 6 | Road and driving conditions | Driving speed | 19 | *“Because of the speed, we were falling forward on the mannequin, and it was very difficult for us to hold the position”* – P13 |
|  |  |  |  | *“The vehicle was moving fast. So, the speed was an issue”* – P08 |
|  |  | Road condition, including humps and Potholes | 16 | *“On turns and on bumps, I lost my balance, and I could have tripped”* – P30 |
|  |  |  |  | *“Because of moving ambulance, because of the roads or if there are any potholes, it will be difficult to give good quality CPR”* – P15 |
|  |  | Traffic | 3 | *“When there is traffic, there will be constant breaks. So, you will keep falling again”* – P15 |
|  |  |  |  | *“Vehicle was moving too fast but, in traffic, during sudden halts, it will be quite challenging to do the CPR”* – P29 |
|  |  | Speed of 40km/h was neutral | 2 | *“Today it was 40 kilometre per hour. It was good because they maintained the right speed”* – P25 |
|  |  |  |  | *“Speed was neutral. The speed was not too slow, not too fast”* – P12 |
| 7 | Ambulance ergonomics | Confined space | 24 | *“There was not enough space to move around”* – P13 |
|  |  |  |  | *“I felt there was less space for me to comfortably stand and perform CPR”* – P10 |
|  |  | Space constraints while switching | 5 | *“When we switch between ventilation and giving CPR in confined space, to move was a challenge”* – P05 |
|  |  | Trolley and chair at the head end were too close | 3 | *“We need more space to stand, because the chair was too close to the trolley, we could not stand properly”* – P16 |
|  |  | Ventilation inside the ambulance | 3 | *“The ventilation was inadequate in the ambulance. It is hot, and you will get dehydrated fast”* – P15 |
|  |  | The bed crank handle was hitting the leg | 2 | *“There was one handle, that was striking my leg”* – P26 |
|  |  | Stretcher height | 1 | *“I feel that if the trolley height was a bit higher, it would have been easy for us to give CPR.”-*P05 |
|  |  | Height of the ambulance | 2 | *“The ceiling of the ambulance was low”* – P29 |
|  |  |  |  | *“Give a better height to the ambulance”* – P13 |
| 8 | Provider safety concerns | Hit head to the shelf | 14 | *“While giving CPR I hit my head twice”* – P02 |
|  |  |  |  | *“Once I hit my head to the front cabin”* – P12 |
|  |  | Injuries to hand from the sharp edges | 2 | *“The cut that I got was from the steel that had no covering on top”* – P28 |
| 9 | Perceived patient safety issues | Patient movement | 1 | *“In moving ambulance, patient also will be moving”* – P16 |
|  |  | Patient physical characteristics-obesity | 1 | *“And the other thing that also plays an important role here is patient's physical appearance. If the patient is very obese, it becomes very difficult to provide CPR”* – P01 |

**Supplemental Table 4: Provider Recommendations to Improve CPR Quality in a Moving Ambulance**

| **Sl No** | **Theme** | **Subtheme** | **Frequency (number of participants)** | **Quote** |
| --- | --- | --- | --- | --- |
| 1 | Physical Readiness and training | Simulation practice added to the curriculum | 20 | *“We can add it to the curriculum and practice it on weekly basis”* – P07 |
|  |  |  |  | *“More simulations in the moving ambulance, so that we get an idea of how to do it”* – P30 |
|  |  | Fitness | 2 | *“Fitness of the EMTs, say if you are not well and have back and neck pain, you won't be able to give high quality CPR in the real life situation”* – P07 |
|  |  | Proper attire | 1 | *“We need to be in a proper attire and comfortable”* – P04 |
| 2 | Human resource and coordination | Have adequate staffing (more than 2 paramedics) | 5 | *“At least 2 paramedics are required to coordinate and perform CPR”* – P02 |
|  |  |  |  | *“It will be very stressful to give CPR in the moving ambulance, sometimes we can get an injury. So more than two people may be helpful to switch and deliver CPR adequately”* – P19 |
|  |  | Supporting colleagues by holding them during turns | 2 | *“One person can hold the one delivering chest compressions when there is a U-turn or a steep turn”* – P04 |
|  |  |  |  | *“The person who was giving the breaths was holding me at my hip, so that during turns we won’t move”* – P21 |
|  |  | Teamwork | 1 | *“Proper teamwork will help improve the quality of the CPR”* – P04 |
| 3 | Balancing techniques | Support from the back (seat/colleague) | 4 | *“If we get any support from the back, we can stand properly to give CPR”* – P18 |
|  |  | Better balance when you widen your legs | 3 | *“We can use some techniques such as widening our legs to keep balance”* – P19 |
|  |  |  |  | *“I kept one leg back and one leg front, then I could give compressions more easily”* – P21 |
|  |  | Wearing shoes (cushioned and sturdier) | 2 | *“The shoes have to be cushioned and sturdier”* – P04 |
|  |  |  |  | *“To prevent fall, maybe we can wear shoes with good grip”* – P05 |
|  |  | Support using knees | 1 | *“We kept our knees bent to give CPR properly”* – P05 |
|  |  | Straddle CPR | 1 | *“Place legs on either side of the patient, that might help”* – P01 |
| 4 | Strategies for Improved Patient Care | Mechanical CPR device | 5 | *“Well, one thing is CPR device, which can be used to have consistent CPR performance”* – P09 |
|  |  | Securing the patient to trolley | 1 | *“A proper locking system for the patient is needed, because the mannequin kept on moving towards us and the wall and the trolley also kept moving”* – P13 |
| 5 | Road Conditions and Ambulance Motion | Good road condition | 3 | *“Other way, we can improve the quality is if the roads are good”* – P15 |
|  |  | Stable driving pattern | 3 | *“If there is smooth driving, we can improve the quality”* – P19 |
|  |  | Speed limit | 3 | *“If the ambulance is going slow, it will be better”* – P18 |
|  |  | Taking slow turns | 1 | *“During the turn, if they take a slow turn, it might help”* – P25 |
| 6 | Ambulance ergonomics modifications | Increase space inside the ambulance | 8 | *“We have less space inside. So, more space is needed for us to stand comfortably”* – P10 |
|  |  |  |  | *“Somehow, if we can increase the space available in the ambulance. Because we will hit each other while switching. So, if you have enough space, we can move easily”* – P03 |
|  |  | Stretcher height higher/ adjustable | 4 | *“Maybe we can heighten the bed accordingly, there should be an option to adjust the height of the bed”* – P10 |
|  |  | Placement of the stretcher in the middle | 2 | *“The placement of the trolley should be central, so that you can switch without having to cross over”* – P25 |
|  |  | Board to kneel and give CPR | 2 | *“I prefer to kneel while performing CPR. So, keeping a board-like surface will help you to improve the quality”* – P27 |
|  |  | Better ventilation | 2 | *“Ventilation is very important because as I said, if you feel suffocated, you cannot provide high quality CPR”* – P15 |
|  |  | Sturdy beds | 1 | *“Patient bed needs to be sturdier”* – P16 |

**Supplemental Table 5: Provider Perspectives on Improving Safety and Comfort of Healthcare Worker during CPR in a Moving Ambulance**

| **Sl No** | **Theme** | **Subtheme** | **Frequency (number of participants)** | **Quote** |
| --- | --- | --- | --- | --- |
| 1 | Balancing techniques | Stabilization devices such as belts/ straps/ suspensions from ambulance | 5 | *“If we have some belt or something to buckle, we will not lose balance and move around”* – P12 |
|  |  |  |  | *“Proper suspensions of the ambulance will help balance better”* – P29 |
|  |  | More handles/ to hold on for balance | 7 | *“Ambulance had one rail to hold. Similarly, if there is one more rail to hold, and if suddenly if they take a break, you can easily hold on to that”* – P05 |
|  |  |  |  | *“Maybe more places to hold on to, such as handles”* – P30 |
|  |  | Grip mat for the floor | 2 | *“May be something that we can grip onto like for example, when you are standing, if there is a grip mat or something, you can stand there with grip”* – P15 |
|  |  |  |  | *“The surface of the ambulance was too slippery. So, our balance was off at that point. For that we can have better floor mats”* – P28 |
|  |  | Training sessions to balance | 2 | *“We can widen our legs, so we can keep balance. It requires practice, so, training sessions to learn balance”* – P23 |
| 2 | Optimized provider ventilation | Ventilation - Fan/AC/ Open windows | 3 | *“It was very clustered, and we were not comfortable, so maybe a fan or an AC would work”* – P10 |
|  |  |  |  | *“Maybe have a fan or a cold air running through the ambulance or open windows, which could allow the air to come in”* – P13 |
| 3 | Ambulance speed control | Speed limit (30-40 km/h) | 3 | *“I guess if you reduce the speed we can give proper CPR. If it is more than 40 or 45km/h, it is a bit of a challenge”* – P03 |
|  |  |  |  | *“Today it was 40 kilometre per hour. It was good. So, maintain the right speed”* – P25 |
| 4 | Ambulance ergonomics | Cushioned shelf, handles and edges | 19 | *“The compartments and the edges could be padded or have a smooth edge”* – P01 |
|  |  |  |  | *“The interior should be padded, so that even if we bang our head or hit our body to the wall of the ambulance, it should be less harmful to us”* – P29 |
|  |  | More space | 10 | *“There should be enough space to stand and also enough space to switch and give CPR”* – P12 |
|  |  |  |  | *“I balanced myself by putting a knee on the stretcher and then give CPR. So maybe if they have more space, I could have gone back and given CPR”* – P06 |
|  |  | Head caps/Padding, Kneecaps for provider joints | 4 | *“Precaution/safety measures to prevent injuries like head caps and elbow caps”* – P14 |
|  |  |  |  | *“Kneecap can be put to prevent injuries”* – P20 |
|  |  | Adjustable height for stretcher | 4 | *“If ambulance patient trolley would be little higher, we could give it more comfortably. It is too low, so we had to bend and give CPR”* – P22 |
|  |  | Move the cabins/ protruding objects to avoid injury | 3 | *“In the ambulance, there are cupboards. There is a chance of getting hit to that. If we can move that and give proper space in the head end, it will be better”* – P24 |
|  |  | Platform to kneel and give CPR | 2 | *“Maybe CPR stand or a platform, where you could kneel and give CPR”* – P06 |
|  |  | Padding on the floor to sit & give CPR | 1 | *“Padding on the floor, if there is adequate spacing and you can sit on the floors and give CPR”* – P13 |
